# An American Physician in Germany

**Published:** 1881-02

**Authors:** 


An American Physician in Germany.—Mr. George Colton, of this city, in one of his letters from Europe, speaks of the reprehensible conduct of the profession in Germany, towards an American, and Marylander, who has thought proper to take up his residence in that country, to practice his profession. He seems to be ignored and ostracised generally, by those to the manner born. Mr. C. speaks of the Marylander as a gentleman, and physician of character, intelligence and culture, and in every way worthy the attention and encouragement of those among whom he has elected to abide.

How strikingly in contrast is the treatment of German physicians in this Country? In fact any one can set up for himself, as a doctor, here, whether he has either brains or culture; and some have succeeded in gulling the people, and making money as physicians, whom, those who knew them at home, declare were engaged in menial or mechanical callings or occupations in their native land. The number of those holding diplomas in this country ought to be sufficiently large to meet all the demands that could be made for doctors without calling upon operatives from shops and workshops of Europe for help! However, our ancestors opened the doors and invited *all* the world to be "free and equal here," but as there is no such reciprocity of action extended from any other quarter, does not the Marylander's case seem to show that the DOORS were opened a trifle too wide?

